# Orexin dual receptor antagonists, zolpidem, zopiclone, eszopiclone, and cognitive research: A comprehensive dose-response meta-analysis

**DOI:** 10.3389/fnhum.2022.1029554

**Published:** 2023-01-09

**Authors:** Mengzhen Zhou, Jiyou Tang, Shasha Li, Yaran Li, Mengke Zhao

**Affiliations:** ^1^Department of Neurology, The First Affiliated Hospital of Shandong First Medical University, Shandong Provincial Qianfoshan Hospital, Jinan, Shandong, China; ^2^Stem Cell Clinical Research Center, National Joint Engineering Laboratory, Regenerative Medicine Center, The First Affiliated Hospital of Dalian Medical University, Dalian Innovation Institute of Stem Cell and Precision Medicine, Dalian, Liaoning, China

**Keywords:** DORA, nBZD, insomnia, cognition, dose-response

## Abstract

**Background:**

About one-third of adults have trouble sleeping, ranging from occasional difficulty to chronic insomnia, along with difficulty maintaining sleep. Many studies reported that the long-term use of hypnotics can cause brain dysfunction and damage cognition.

**Objective:**

The objective of the study is to evaluate whether low, medium, and high doses of orexin dual receptor antagonists (DORA), zopiclone (ZOP), eszopiclone (ESZ), and zolpidem (ZST) can impair cognition.

**Methods:**

From the beginning through September 20, 2022, PubMed, Embase, Scopus, the Cochrane Library, and Google Scholar were searched. Randomized controlled trials (RCTs) assessing the therapeutic effects of DORA, eszopiclone, and zopiclone for sleep and cognitive function were included. The primary outcomes were indices related to the cognitive profile, including memory, alertness, execution and control function, and attention and orientation. The secondary outcomes were indices related to sleep and adverse events. The standard mean difference (SMD) was generated for continuous variables. Certain data were captured from figures by GetData 2.26 and analyzed using RStudio 4.2.

**Results:**

Finally, a total of 8,702 subjects were included in 29 studies. Compared with the placebo, the DSST (Digit Symbol Substitution Test) scores of low, medium, and high doses of DORA were SMD = 0.77; 95% CI: 0.33–1.20; SMD = 1.58; 95% CI: 1.11–2.05; and SMD = 0.85; 95% CI: 0.33–1.36, respectively. The DSST scores of zolpidem at low, medium, and high doses were SMD = −0.39; 95% CI: 0.85–0.07; SMD = −0.88, 95% CI: −2.34–0.58; and SMD = −0.12, 95% CI: −0.85–0.60, respectively. Zopiclone's DSST scale score was SMD = −0.18; 95% CI: −0.54–0.18. In addition, the total sleep time (TST) of low, medium, and high doses of DORA was SMD = 0.28, 95% CI: −0.15–0.70; SMD = 1.36, 95% CI: 0.87–1.86; and SMD = 2.59, 95% CI: 1.89–3.30, respectively. The TST of zolpidem with low, medium, and high doses was SMD = 1.01, 95% CI: 0.18–1.83; SMD = 1.94, 95% CI: 0.46–3.43; and SMD = 1.71, 95% CI: 0.86–2.56, respectively. The TST of low, medium, and high doses of eszopiclone was relatively SMD = 2.03, 95% CI: −0.21–4.27; SMD = 2.38, 95% CI: 1.35–3.42; and SMD = 1.71, 95% CI: 0.60–2.82. Zopiclone's TST was SMD = 2.47, 95% CI: 1.36–3.58.

**Conclusion:**

We recommend DORA as the best intervention for insomnia because it is highly effective in inducing and maintaining sleep without impairing cognition. Although zolpidem has a more pronounced effect on maintaining sleep, it is best to reduce its use because of its side effects. Eszopiclone and zopiclone improved sleep quality, but their safety in cognition remains to be verified.

## Introduction

In people aged over 18 years old, the overall prevalence of insomnia is about 32.2% worldwide, according to epidemiological studies. Moreover, according to statistics of annual amount of sleeping drugs used in China, which was provided by Food and Drug Administrations of China, it was found that the general Chinese population consumes around 339 million tablets of hypnotic medications annually, with zolpidem ranked first as the top hypnotic Adjacent to references (De Gage et al., 2014; Picton et al., 2018). Don't distinguish front and rear positions drug with 133.7 million pills (Foda and Ali, 2012; Julie and Dopheide, 2020; Westermeyer and Carr, 2020). Brain function changes during sleep loss, including altered cognitive function in brain regions involved in perceptual abilities (alertness and orientation), attention, memory, and executive control (Zhang et al., 2018; Chao et al., 2021).

Cognition is the behavior in which the brain receives information from the outside world and processes it into intrinsic mental activity (Haynes et al., 2018). People are concerned about damaging cognition when taking hypnotic drugs, which causes poor concentration, memory decline, and so on (Sulheim et al., 2015; Olaithe et al., 2018; Suchting et al., 2020). Sleep and cognitive function are closely related. Effective sleep aids should maximize the patient's perception of sleep quality and avoid drug-related adverse reactions without altering the underlying structural characteristics of sleep (Aarsland, 2016). As a novel hypnotic drug, DORA inhibits patients' excessive wakefulness and induces and maintains sleep by antagonizing the orexin signaling system (Sakurai, 2013). Although animal experiments have pointed to a slight cognitive-promoting effect of low-dose DORA, there have been no clinical studies or meta-analyses to indicate whether the use of DORA affects cognitive function in subjects (Hoyer and Jacobson, 2013; Gamble et al., 2020; Zhou et al., 2020). This study divided four commonly used hypnotics into low, medium, and high dosages. Their efficacy and safety were comprehensively analyzed in terms of aspects of sleep, cognition, and adverse effects to provide a reference for using hypnotics.

## Methods

This meta-analysis was performed following the PRISMA guidelines (Preferred Reporting Items for Systematic Reviews and Meta-analysis) (Page et al., 2021). The review scheme is registered with PROSPERO, the International Prospective System Review Register (Unique Identifier: CRD42022352911).

### Search strategy

The literature was retrieved from independent databases, including PubMed, Embase, Scopus, the Cochrane Library, and Google Scholar. The Mesh terms were (“orexin dual receptor antagonist” OR “Suvorexant” OR “Filorexant” OR “Lemborexant” OR “Almorexant” OR “Daridorexant” OR “sb 649868”) AND (“Stilnox” OR “zolpidem”) AND (“Eszopiclone” OR “eszopiclone”) AND (“Zopiclone” OR “zopiclone”) AND (“Sleep” OR “sleep” OR “sleeping”) AND (“Cognitive function” OR “cognitive function;” OR “cognitive functions” OR “cognitional function” OR “Cognitive functioning” OR “Cognition function”).

### Inclusion and exclusion criteria

#### Inclusion criteria

1. Cohort studies, case-control studies, and randomized controlled studies (RCTs) encompassing both subjects without insomnia and those with insomnia;
2. Cohort studies, case-control studies, and randomized controlled studies (RCTs) encompassing both subjects who are non-cognitively impaired and subjects with cognitively impaired;
3. Cohort studies, case-control studies, and randomized controlled studies (RCTs) using at least one of the following agents: dual orexin receptor antagonists: suvorexant, filorexant, lemorexant, almorexant, daridorexant, sb 649868, and zolpidem. The dosages of these drugs should be definite.

#### Exclusion criteria

1. The agents including orexin dual receptor antagonists or zopiclone or eszopiclone or zolpidem were in combination with other hypnotic drugs in the treatment group.
2. Repeated publication of literature.
3. Unable to extract data or data were unclear or missed in the literature.
4. Conference proceedings.
5. Non-RCT articles.

### Data extraction and quality assessment

Two investigators independently searched, screened, assessed the quality, and extracted information from articles. The following data were collected: the number of subjects, age, sex, race, drugs and corresponding dosages, cognitive outcomes, sleep outcomes, and adverse events. Any discrepancy was arbitrated by a senior investigator.

### Outcomes

The primary outcomes were indices related to the cognitive profile, including memory, alertness, execution and control function, attention and orientation, etc.

The secondary outcomes were indices related to sleep and adverse events.

For RCTs, risk of bias tools (second edition, ROB2), according to the Cochrane Handbook, were used to assess the quality, while for cohort and case-control studies, the Newcastle-Ottawa Scale (NOS) was performed to assess the quality (Stang, 2010).

### Definition of various doses

High dose: zolpidem ≥ 10 mg, eszopiclone = 3 mg, Suvorexant ≥ 15 mg, filorexant ≥ 15 mg, lemborexant > 10 mg, almorexant ≥ 30 mg, daridorexant > 25 mg, and SB-649868 > 30 mg.

Moderate dose: 5 mg < zolpidem < 10 mg, eszopiclone = 2 mg,10 mg ≤ suvorexant < 15 mg, 10 mg ≤ filorexant < 15 mg, 5 mg < lemborexant ≤ 10 mg, 10 mg ≤ almorexant ≤ 30 mg, 10 mg ≤ daridorexant ≤ 25 mg, and 10 mg < SB-649868 ≤ 30 mg.

Low dose: zolpidem ≤ 5 mg, eszopiclone = 1 mg, suvorexant < 10 mg, filorexant < 10 mg, lemborexant ≤ 5 mg, almorexant < 10 mg, daridorexant < 10 mg, and SB-649868 ≤ 10 mg.

### Statistical methods

The software utilized was RStudio 4.2.1. The standard mean difference (SMD) was generated as the effect size for continuous variants. Odds ratios (ORs) were generated for dichotomous variants. If only figures were presented, two researchers independently used GetData 2.26 to capture data and calculate the means. A fixed-effects model would be implemented if *I*^2^ ≤ 50% and *p* > 0.01. Otherwise, a random-effects model would be performed. If *I*^2^ > 75%, Galbraith plots would be drawn to exclude studies outside the outlines to eliminate heterogeneity. Publication bias was assessed by the funnel plot and Egger's test. A probability value of *p* < 0.05 was considered statistically significant.

## Results

### Study search and baseline characteristics and quality

**Figure 1:**
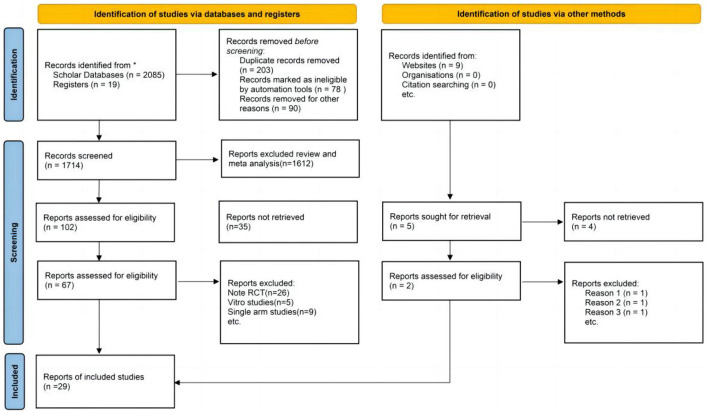
Preferred reporting items for systematic reviews and meta-analyses (PRISMA) flow diagram of study search and selection for the meta-analysis.

In the preliminary phase, a total of 2,085 articles were searched, and after careful screening, 27 RCTs and 2 case-control studies were eventually considered eligible for analysis (Figure 1) (Ancoli-lsrael, 2010; McCall et al., 2010; Menza et al., 2010; Mets et al., 2011; Bettica et al., 2012; Herring et al., 2012, 2013, 2014, 2016, 2017, 2020; Hoever et al., 2012, 2013; Uchimura et al., 2012a,b; Sun et al., 2013; Leufkens et al., 2014; Tek et al., 2014; Vermeeren et al., 2014, 2016, 2019; Spierings et al., 2015; Connor et al., 2016; Dauvilliers et al., 2020; Dopheide, 2020; Karppa et al., 2020; Murphy et al., 2020; Zammit et al., 2020; Bland et al., 2021; Boof et al., 2021; Louzada et al., 2022). The subjects of these studies were from America, Australia, Britain, and Germany, among others. Their ages ranged from 18 to 85 years, and the proportion of women was 47.3%.

The DORA agents include suvorexant, filorexant, lemborexant, almorexant, daridorexant, and SB-649868. According to ROB2, all 27 RCTs were of high quality. The two case-control studies had a NOS score of 4.

### Cognitive profile—Memory

**Figure 2:**
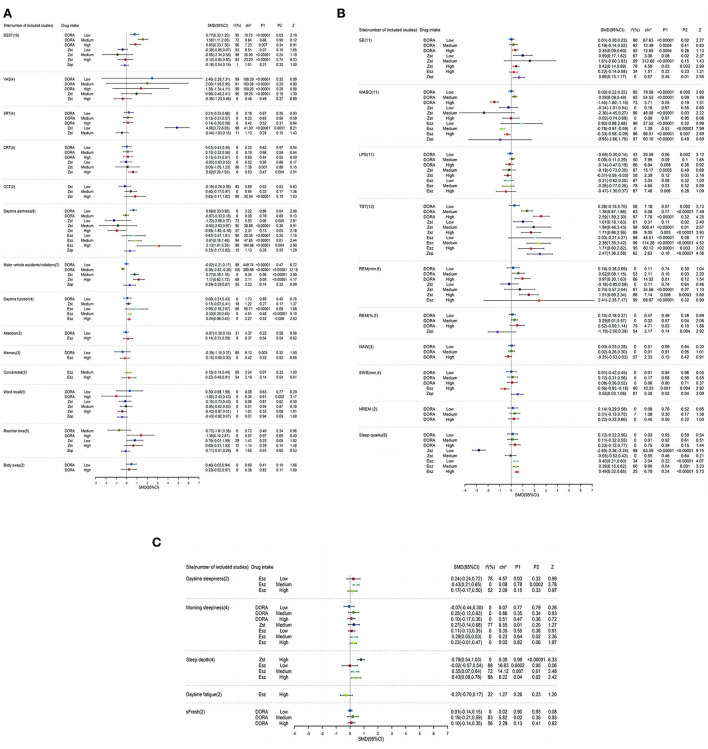
**(A–C)** A Forest map of cognition and sleep results at different doses in DORA, zolpidem, zopiclone, and eszopiclone groups. DSST, Digit symbol substitution test; VAS, visual analog scale; SRT, simple response time scale; CCT, competitive cognitive trait anxiety inventory; CRT, competency ratio table; SE, Sleep efficiency; WASO, wake after sleep onset; LPS, latency to persistent sleep; TST, total sleep time; REM, rapid eye movement; NAW, number of awakenings; SWS, slow wave sleep; NREM, non-REM sleep.

**Figure 3:**
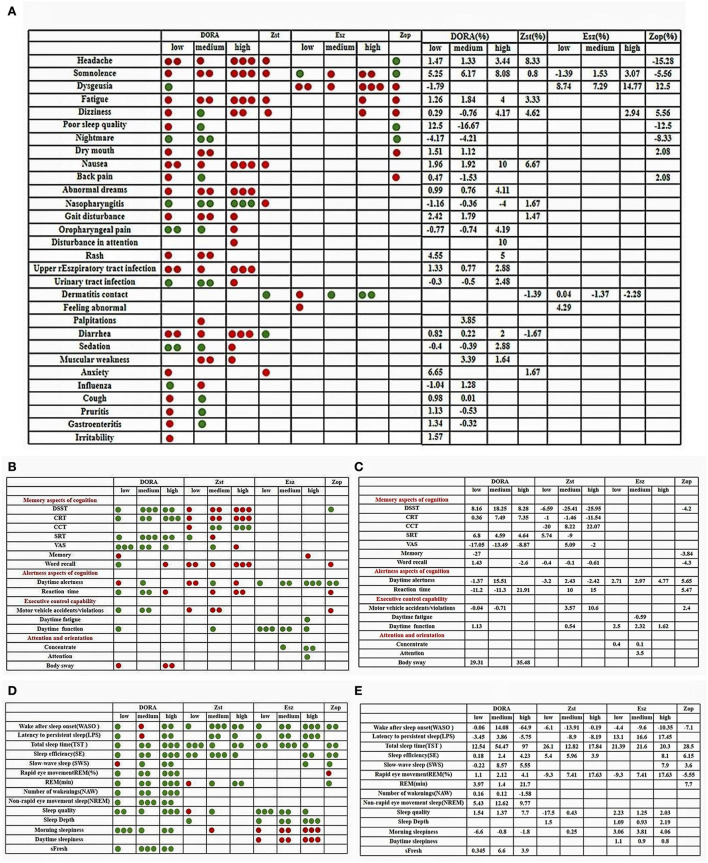
**(A–E)** Color contrast quality evaluation chart. The evaluation method of each related index is to evaluate the clinical treatment effect of the subject. The green point is to improve and have a positive effect. The red point is to damage, reduce, and produce negative harm. The number of points is evaluated according to the numerical value. The larger the numerical value, the more the number of points.

**Figure 4:**
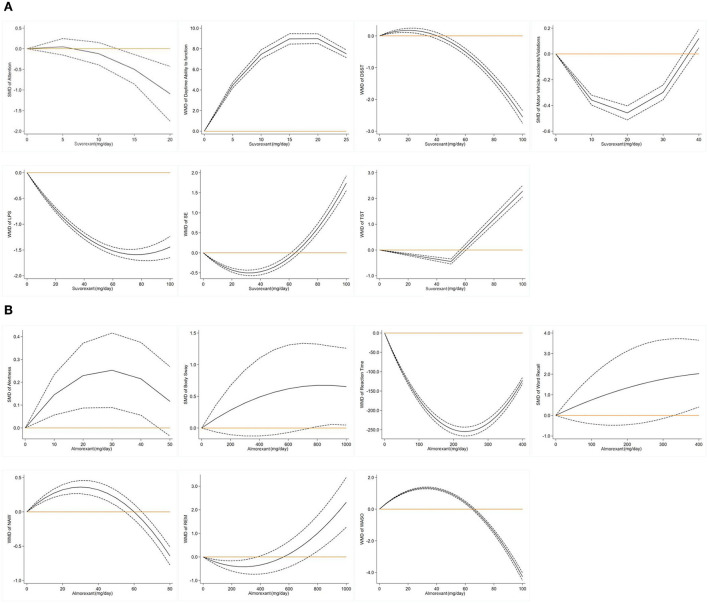
**(A)** Suvorexant is the first approved drug under DORA. According to the dose-response non-linear curve, at 20 mg, the subjects scored the highest on the DSST scale, and the incidence of motor vehicle driving violations/accidents was the lowest. At doses below 20 mg, the subjects had better information processing, attention, and psychomotor abilities. The diurnal function of the subjects in the suvorexant group decreased significantly after taking a dose of more than 20 mg. It was concluded that the maximum dose of suvorexant should not be more than 20 mg, which was consistent with the recommended doses of 5, 10, 15, and 20 mg in the guidelines. The sleep result seems to have little relationship with the dose. With the increase in dose, LPS decreases, and TST prolongs. SE is lower when it is <30 mg, then SE increases. **(B)** In the Almorexant group, the subjects' alertness scores were higher at 10 mg to 30 mg and reached the highest alertness at 30 mg, while when the dose was >30 mg, the alertness gradually decreased. In addition, the reaction time decreased with the increase in dose. The dose did not affect the word memory score and body swing amplitude. NAW and WASO showed an upward trend before 30 mg and reached the highest point at 30 mg.

Compared with the placebo group, the memory test score of low-dose DORA was SMD = −0.39, 95% CI: −1.15–0.37 (Figures 2–4). A memory test score of high-dose eszopiclone was SMD = −0.15, 95% CI: −0.60–0.30. The DSST (Digit Symbol Substitution Test) scale scores of low, medium, and high doses of DORA were SMD = 0.77, 95% CI: 0.33–1.20; SMD = 1.58, 95% CI: 1.11–2.05; and SMD = 0.85, 95% CI: 0.33–1.36, respectively. The DSST scale scores of zolpidem at low, medium, and high doses were, respectively, SMD = −0.39, 95% CI: −0.85–0.07; SMD = −0.88, 95% CI: −2.34–0.58; and SMD = −0.12, 95% CI: = 0.85–0.60. Zopiclone's DSST scale score was SMD = −0.18, 95% CI: = 0.54–0.18. The DSST scale is the most widely used cognitive screening scale, which can comprehensively and quickly reflect the intellectual status and cognitive function decline of the person being tested. It is also a part of the revised Wechsler Adult Intelligence Scale, which assesses information processing, attention, and psychomotor performance.

DORA doses in each group have, respectively, improved the total score. Zopiclone and zolpidem low-dose groups have helped in SRT (simple response time scale) and VAS (visual analog scale). The subjects taking medium and high doses of ZST benefit from improving their CCT (Competitive Cognitive Trait Anxiety Inventory) scale score and do not affect other doses. The SRT was used in both memory research and clinical research, and the selective response time scale CRT (Competency Ration Table), which is famous for detecting the residual effects of hypnotics like medium-dose DORA, are superior to those used in the placebo group in terms of total score or single item of the scale (Miller et al., 1989; Stormer et al., 2012).

The word recall accuracy score of low-dose/high-dose DORA was SMD = 0.30, 95% CI: −0.98–1.58 and SMD = −1.00, 95% CI: −2.43–0.43, respectively. The correct rate score of word recall of zolpidem at low, medium, and high doses was SMD = −0.15, 95% CI: −0.73–0.43; SMD = −0.05, 95% CI: −0.62–0.53; and SMD = −0.43, 95% CI: −0.87–0.01, respectively. Zopiclone's Word recalls accuracy score was SMD = −0.43, 95% CI: −0.92–0.07. The memory was evaluated before and 4 h after administration (measured by word recall). Compared with the placebo group, the number of correct words in almost every dose of ZST decreased, which was unrelated to the dose. The low dose of DORA significantly increased the number of correct words recalled.

### Cognitive profile—Alertness

Compared with the placebo group, the daytime alertness score of low-dose and middle-dose DORA was SMD = 0.66, 95% CI: 0.33–0.98 and SMD = −0.07, 95% CI: −0.33–0.18, respectively. The daytime alertness score of zolpidem at low, medium, and high doses was SMD = −1.22, 95% CI: −2.06–0.37; SMD = −0.83, 95% CI: −2.63–0.97; and SMD = −0.95, 95% CI: −1.80–0.10, respectively. The daytime alertness score of low, medium, and high dose eszopiclone was SMD = 0.67, 95% CI: −0.47–1.81; SMD = 0.81, 95% CI: 0.16–1.46; and SMD = 3.13, 95% CI: 1.01–5.25, respectively. Zopiclone's daytime alertness score was SMD = 0.33, 95% CI: −0.17–0.82. There are some differences between DORA and zolpidem. DORA causes a dose-dependent decrease in subjective alertness (reaction time), which is more obvious than zolpidem. In this test, compared with the better-performing eszopiclone and zopiclone groups, zolpidem had no significant therapeutic effect on motor coordination. In the morning, the single dose of low-dose, middle-dose, and high-dose DORA has a statistically significant impact on the “wake up mode” and “wake up behavior” after taking it, compared with the placebo group, indicating that it is more difficult to wake up at a high dose and that alertness after waking up is lower. However, the overall therapeutic effect of low- and medium-dose DORA on alertness is better than that of the placebo group, which will improve alertness.

The reaction time of medium and high doses of DORA was, respectively, SMD = 0.72, 95% CI: 1.81–0.38 and SMD = 1.39, 95% CI: 0.10–2.67. Low- and high-dose zolpidem's reaction time was SMD = 0.79, 95% CI: 0.01–1.59 and SMD = 0.65, 95% CI: 0.21–1.52, respectively. Zopiclone's reaction time was SMD = 0.11, 95% CI: 0.51–0.29. Psychomotor performance was measured by the selection of the reaction time. DORA reduced the selective reaction time by 1.5, 4, and 8 h after administration, but the effect was not obvious. This indicates that DORA does not damage situational memory after waking up in the middle of the night.

### Cognitive profile—Execution and control function

When taking zopiclone and a low and medium dose of zolpidem, the driving ability of the subjects was impaired. Users who needed to drive the next day or perform tasks that required full attention had to be warned and were asked to be mindful of the possibility of an accident/violation while driving the next day. The standard deviation of the lateral position (SDLP cm) in the standardized road driving test evaluated the driving performance. We evaluated individual-level driving performance to measure whether there was a statistically significant imbalance between drug placebo differences and relative driving impairment. Compared with the placebo group, the incidence of motor vehicle accidents/violations with low- and middle-dose DORA was SMD = 0.02, 95% CI: 0.21–0.17; SMD = 0.36, 95% CI: 0.52–0.20, respectively. The incidence of motor vehicle accidents/violations with medium- and high-dose zolpidem was SMD = 0.77, 95% CI: 0.39–1.16 and SMD = 1.17, 95% CI: 0.62–1.72, respectively. Zopiclone's motor vehicle accident/violation rates were SMD = 0.29, 95% CI: 0.29–0.87). Compared with zolpidem, low- and medium-dose DORA improves some aspects of the cognitive performance of motor vehicle driving.

The daytime function scores were SMD = 0.06, 95% CI: 0.31–0.43 in low-dose DORA group, and SMD = 0.17, 95% CI: 0.07–0.41 in medium dose zolpidem group. The daytime function scores of low/medium/high dose Eszopiclone were SMD = 0.95, 95% CI: −0.16–2.07; SMD = 0.33, 95% CI: 0.20–0.45; SMD = 0.24, 95% CI: 0.06–0.42, respectively. After taking sleeping pills, subjects were asked to be weary of any sloppiness, slowness in action, and poor reaction time when driving the next day; it could have been dangerous. The results showed that DORA and eszopiclone also improved the daytime function of people with insomnia with good safety.

### Cognitive profile—Attention and orientation

Balance and psychomotor performance were assessed during the night. Balance (body sway as measured by platform stability) was assessed before and 1.5, 4, and 8 h after administration. Compared with the placebo group, low-dose DORA's attention score was SMD = −0.07, 95% CI: −0.30–0.16. The intensity score of high-dose eszopiclone was SMD = 0.14, 95% CI: −0.31–0.59. The concentration scores of medium and high-dose eszopiclone were SMD = 0.15, 95% CI: −0.14–0.44; SMD = 0.22, 95% CI: −0.48–0.91, respectively.

The body sway scores of low-dose and high-dose DORA were SMD = 0.46, 95% CI: −0.03–0.94; SMD = 0.23, 95% CI: −0.52–0.97, respectively. In all DORA doses, the overall therapeutic effect on body sway was not as good as that in the placebo group, and body sway increased (injury). The stability when getting up (for example, using the bathroom) was poor. These results suggest that DORA may be a potential risk, especially for elderly patients who are prone to falling and moving slowly. The subjective assessment showed that the decrease in motor coordination was dose-dependent. In the middle dose, it significantly reduced the motor coordination ability. Each dose of Eszopiclone improved attention so that the subjects could concentrate more on clearly perceiving certain things and thinking deeply about certain problems without disturbance.

### Sleep profile

In general, the sleep effect of each group was definite, and the response was good. DORA, zolpidem, zopiclone, and eszopiclone can shorten subjective and objective sleep latency (LPS), reduce the number of awakenings (NAW), extend the total sleep time (TST), and can induce sleep quickly. To some extent, it can prolong the sleep phase, deepen sleep, and improve sleep quality. In addition, it is remarkable that many studies found that DORA does not seem to significantly change the underlying sleep structure characteristics. In addition to the subjective and objective effects on the beginning and maintenance of sleep, DORA also improves the patients' perception of sleep quality. Subjectively, it significantly improves the emotional improvement and the person feels refreshed in the morning. The Self fresh score of low, medium, and high doses of DORA group was SMD = 0.01, 95% CI: −0.14–0.15; SMD = 0.19, 95% CI: −0.21–0.59; and SMD = 0.10, 95% CI: −0.14–0.35, respectively. The number of awakenings after falling asleep (WASO) in each comfort group was higher than in the experimental group. The WASO of low, medium, high doses of DORA was SMD = 0.00, 95% CI: −0.22–0.22; SMD = 0.29, 95% CI: 0.09–0.49; and SMD = −1.45, 95% CI: −1.80–1.10, respectively. The WASO of low, medium, and high doses of zolpidem was SMD = 0.24, 95% CI: 1.01–0.54; SMD = 2.36, 95% CI: 4.45–0.27; and SMD = 0.02, 95% CI: 0.74–0.69, respectively. The WASO of low, medium, and high doses of eszopiclone was SMD = 0.90, 95% CI: −0.88–2.68; SMD = −0.78, 95% CI: −0.97 to −0.59; and SMD = −0.33, 95% CI: −0.58 to −0.09. The WASO of zopiclone was SMD = 0.95, 95% CI: 3.66–1.76).

Slow wave sleep (SWS) performance in the placebo group was also inferior to that in the sleeping drug group. Slow-wave sleep plays an important role in restoring physical energy and growth. The SWS of low, medium, and high doses of DORA were SMD = 0.01, 95% CI: −0.42–0.45; SMD = 0.12, 95% CI: −0.31–0.56; and SMD = 0.08, 95% CI: −0.36–0.52, respectively. The SWS of high-dose eszopiclone was SMD = −0.56, 95% CI: −0.93 to −0.18. The SWS of zopiclone was SMD = 0.55, 95% CI: 0.03–1.06. In addition, the rapid eye movement sleep (REM) of the ZST group also decreased. The study found that, during REM sleep, brain protein synthesis was accelerated, and oxygen consumption and blood flow were increased, which could promote the recovery of energy and memory storage, thus improving the learning and working efficiency of the next day.

Sleep efficiency (SE) of low, medium, and high doses of DORA were SMD = 0.01, 95% CI: −0.20–0.23; SMD = 0.19, 95% CI: −0.14–0.52; and SMD = 0.35, 95% CI: 0.09–0.60, respectively. The SE of zolpidem at low, medium, and high doses were SMD = 0.99, 95% CI: 0.17–1.82; SMD = 1.61, 95% CI: −0.60–3.83; and SMD = 0.42, 95% CI: 0.14–0.69. The SE of high dose of eszopiclone (SMD = 0.22, 95% CI: −0.14–0.58). The SE of zopiclone was SMD = 0.66, 95% CI: 0.15–1.17. Low sleep efficiency often induces headaches, dizziness, tension, anxiety, and other behaviors, and even falls due to muscle weakness. Several hypnotics improve sleep efficiency to a certain extent.

### Adverse event profile

**Figure 5:**
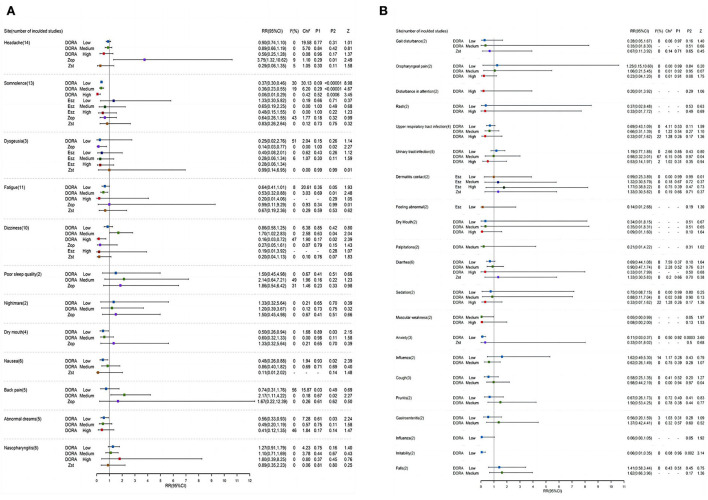
**(A,B)** A Forest map of adverse reactions in DORA, zolpidem, zopiclone, and eszopiclone groups.

Compared with the placebo group, the probabilities of low, middle, and high doses of DORA to develop headache were RR = 0.90, 95% CI: 0.74–1.10; RR = 0.89, 95% CI: 0.66–1.19; and RR = 0.56, 95% CI: 0.25–1.28. Zolpidem's probability of causing a headache was RR = 0.29, 95% CI: 0.06–1.35 (Figure 5). The probability of zopiclone causing a headache was RR = 3.75 (95% CI: 1.32–10.62). The incidence of adverse events was analyzed by the RR method. According to the forest map of adverse results, the incidence of adverse reactions among subjects in the DORA, zolpidem, zopiclone, and eszopiclone groups was higher than that in the placebo group. The most common adverse reactions were headache and somnolence. The probability of somnolence in low, medium, and high doses of DORA was RR = 0.37, 95% CI: 0.30–0.46; RR = 0.36, 95% CI: 0.23–0.55; and RR = 0.06, 95% CI: 0.01–0.29, respectively. The probability of somnolence with zolpidem was RR = 0.83, 95% CI: 0.26–2.64. The probability of somnolence with low, medium, and high doses of eszopiclone was relatively low: RR = 1.33; 95% CI: 0.30–5.82; RR = 0.65, 95% CI: 0.19–2.25; and RR = 0.48, 95% CI: 0.15–1.55, respectively. The probability of somnolence with zopiclone was RR = 0.64, 95% CI: 0.26–1.55. In addition, the probability of fatigue occurring in low, medium, high doses of DORA was RR = 0.64, 95% CI: 0.41–1.01; RR = 0.53, 95% CI: 0.32–0.88; and RR = 0.20, 95% CI: 0.01–4.06, respectively. The probability of zolpidem's fatigue was RR = 0.67, 95% CI: 0.19–2.36. The probability of zopiclone fatigue was RR = 0.99, 95% CI: 0.11–9.29. The present study's most common adverse events included somnolence, headache, dizziness, abnormal dreams, fatigue, diarrhea, nausea, and dry mouth. The incidence of adverse reactions was reported more at high doses than at low doses. No serious adverse reactions were reported, and the drug reactions were good.

## Discussion

As far as we know, this meta-analysis is the first dose-response meta-analysis of DORA cognitive research. DORA is considered to be the latest insomnia treatment method. The study found that it would not damage the sleep structure, and its slight cognitive promotion effect in animal experiments has become an undisputed research focus in the field of sleep disorders in recent years. In the present study, we proved that, compared with the placebo group, DORA has a lower incidence of adverse reactions, such as headache (1.47 and 1.33% increase, respectively, in low and medium doses), lethargy (5.25 and 6.17% increase in low and medium doses, respectively), and fatigue (1.26 and 1.84% increase, respectively, in low and medium doses), which can effectively improve the perception of sleep quality of subjects. Consistent with many studies, subjects report feeling refreshed the next morning. The subjects were energetic (low, medium, and high doses increased the sFresh score by 0.345, 6.6, and 3.9 points, respectively) and suffered no damage to the daytime function (SMD = 0.06, 95% CI = 0.31–0.43) or memory performance (SMD = −0.39, 95% CI −1.15–0.37), showing superior efficacy and safety. Although zolpidem (low dose) is very effective in shortening sleep latency (SMD = −0.19, 95% CI = 0.73–0.35) and prolonging sleep duration (SMD = 1.01, 95% CI = 0.18–1.83), its tolerance and safety are not ideal. It is likely to damage the memory and alertness of the subjects and increase the risk of motor vehicle driving violations/accidents the next day. Eszopiclone (low dose) played an active role in daytime sensitivity (SMD = 0.67, 95% CI = 0.47–1.81) and daytime function (SMD = 0.95, 95% CI = 0.16–2.07). Surprisingly, the incidence of headaches in the zopiclone group was 15.28% lower than in the control group. The incidence of nightmares decreased by 8.33%.

Over the years, DORAs has developed into a very successful hypnotic drug. DORA inhibits the hyperactive arousal pathway of people with insomnia by blocking orexin signal transduction. The effect of an ideal hypnotic is to fall asleep quickly, have enough sleep throughout the night, and have a reduced residual sleepy effect the next morning. DORA benefits people by improving sleep, enhancing metabolic waste removal, and strengthening the circulation of the glial lymphoid system (Peleg-Raibstein and Burdakov, 2021). In the follow-up survey, DORA was well tolerated without serious safety problems, rebound, or withdrawal reactions (Scott, 2020; Esmaili-Shahzade-Ali-Akbari et al., 2021). Based on the different aspects of the cognitive field, orexin can directly project to the hippocampus and affect learning and memory and regulate cognition, indicating that DORA treatment may be a potential strategy to improve the early cognitive impairment of patients with insomnia (Kukkonen, 2017). Non-benzodiazepines (NBZDs), which include zolpidem, zopiclone, and eszopiclone, have fewer addictive effects, fewer neuromuscular effects, and fewer damage to cognitive function. Especially zolpidem and eszopiclone, which only agonize hypnotic receptors, do not aggravate receptors involved in muscle relaxation, anxiolysis, and cognitive impairment, NBZDs are less disruptive to normal sleep architecture, are safer than benzodiazepines (BZDs), and have less daytime sedation and other adverse effects (Nielsen, 2017). However, as a first-line therapeutic drug in clinical application, the cognitive results of zolpidem are not satisfactory. In addition, there is no clear “gold standard” for measuring hypnotic drugs and cognitive results. Zopidem has minor sequelae, tolerance, drug dependence and withdrawal symptoms, and a wide range of safety (Holm and Goa, 2000). However, when used with other central inhibitors, it can cause severe respiratory depression (Castro et al., 2020). It is suitable for occasional and temporary insomnia. It is a short-term sleeping drug with common adverse reactions such as hallucinations, excitement, nightmares, and depression. Zopiclone and eszopiclone are representatives of the third generation of sedative-hypnotic drugs (Zhang et al., 2020). These drugs have definite efficacy, few adverse reactions, rapid action, and effectiveness for up to 6 h, enabling patients to fall asleep quickly and maintain sufficient sleep depth (Terzano et al., 2003). No obvious drug resistance was found after long-term use, and no rebound was found after drug withdrawal (Hesse et al., 2003; Stranks and Crowe, 2014). The latest drug zopiclone is a dextral isomer of zopiclone. The latest drug dexzopiclone is a dextro isomer of zopiclone and is two times more potent than the parent but less toxic than the parent. The order of addiction level is as follows: benzodiazepines > zopiclone > zolpidem.

We can assume that DORA is used as a potential preventive, therapeutic, or neuroprotective drug to target the downregulation of the orexinergic system (Kumar et al., 2016), not only to manage the sleep disorder of patients with insomnia but also to improve sleep to slow down their neurodegenerative process and slow down their cognitive impairment (Drake et al., 2019). In short, taking DORA may provide a new possibility for treating insomnia (mainly mild to moderate) by improving sleep and enhancing cognition (Roch et al., 2021).

Evaluating the cognitive effects of hypnotics is a complex and challenging task. First, due to limited amount of the extended timespan of the hypnotics given in the articles selected for our study and the small number of literature available, more research is needed on the residual effects of clinically relevant doses. Second, considering that DORA only slightly improves short-term memory, there is no evidence that it can help improve long-term memory, and the study duration of the included trials was relatively short (Seol et al., 2019; Toor et al., 2021). It is necessary to extend the experimental time and follow-up regularly. Finally, because everyone has a different sensitivity to this drug, we should also consider the effect of treatment. In short, in addition to high-quality randomized controlled trials, more research is needed to solve the problem of combining DORA with cognition in clinical practice to help more patients.

## Author contributions

JT participated in the topic selection design of the article. MZhou wrote the article. SL, YL, and MZhao revised the article. All authors contributed to the article and approved the submitted version.

## Funding

This work was supported by National Natural Science Foundation of China (Exploring the molecular and cellular mechanism of sleep deprivation impairing learning and memory based on orexin-A signal transduction characteristics, 81471345), Shandong Natural Science Foundation Project (Research on the effect of sleep disturbance on learning and memory in AD and its molecular and pathological mechanism, ZR2020mh160), and Horizontal Project of Shandong University (control study of early intervention of sleep disorder on delaying Alzheimer's disease, 089/2019).

## Conflict of interest

The authors declare that the research was conducted in the absence of any commercial or financial relationships that could be construed as a potential conflict of interest.

## Publisher's note

All claims expressed in this article are solely those of the authors and do not necessarily represent those of their affiliated organizations, or those of the publisher, the editors and the reviewers. Any product that may be evaluated in this article, or claim that may be made by its manufacturer, is not guaranteed or endorsed by the publisher.
